# Correlation of Increased Blood Levels of GITR and GITRL with Disease Severity in Patients with Primary Sjögren's Syndrome

**DOI:** 10.1155/2013/340751

**Published:** 2013-07-07

**Authors:** Xiaoxia Gan, Xiaoke Feng, Lei Gu, Wenfeng Tan, Xiaoxuan Sun, Chengyin Lv, Miaojia Zhang

**Affiliations:** Department of Rheumatology, The First Affiliated Hospital of Nanjing Medical University, 300 Guangzhou Road, Nanjing 210029, China

## Abstract

Glucocorticoid-induced tumor necrosis factor receptor family-related protein (GITR) is a type I transmembrane protein belonging to the TNFR superfamily. After activated by its ligand GITRL, GITR could influence the activity of effector and regulatory T cells, participating in the development of several autoimmune and inflammatory diseases included rheumatoid arthritis and autoimmune thyroid disease. We previously reported that serum GITRL levels are increased in systemic lupus erythematosus (SLE) patients compared with healthy controls (HC). Here, we tested serum soluble GITR (sGITR) and GITRL levels in 41 primary Sjögren's syndrome (pSS) patients and 29 HC by ELISA and correlated sGITR and GITRL levels with clinical and laboratory variables. GITR and GITRL expression in labial salivary glands was detected by immunohistochemistry. pSS patients had significantly increased serum levels of sGITR and GITRL compared with controls (GITR: 5.66 ± 3.56 ng/mL versus 0.50 ± 0.31 ng/mL; *P* < 0.0001; GITRL: 6.17 ± 7.10 ng/mL versus 0.36 ± 0.28 ng/mL; *P* < 0.0001). Serum sGITR and GITRL levels were positively correlated with IgG (GITRL: *r* = 0.6084, *P* < 0.0001; sGITR: *r* = 0.6820, *P* < 0.0001) and ESR (GITRL: *r* = 0.8315, *P* < 0.0001; sGITR: *r* = 0.7448, *P* < 0.0001). Moreover, GITR and GITRL are readily detected in the lymphocytic foci and periductal areas of the LSGs. In contrast, the LSGs of HC subjects did not express GITR or GITRL. Our findings indicate the possible involvement of GITR-GITRL pathway in the pathogenesis of pSS. Further studies may facilitate the development of targeting this molecule pathway for the treatment of pSS.

## 1. Introduction

Glucocorticoid-induced tumor necrosis factor receptor family-related protein (GITR) is a type I transmembrane protein belonging to the TNFR superfamily, which was originally discovered by Nocentini et al. as a gene upregulated in dexamethasone-treated murine T cell hybridomas [1]. GITR has low basal expression on naive murine CD4^+^ and CD8^+^ T cells as well as human T cells, similar to other TNFR family members such as 4-1BB and OX40 [2–5]. However, both murine and human regulatory T cells (Tregs) constitutively express GITR [6]. GITR is activated by its ligand GITRL (TNFSF18), a type II transmembrane protein belonging to the TNF superfamily. GITRL is expressed at low levels by antigen-presenting cells such as macrophages, dendritic cells, and B cells and is upregulated upon activation, consistent with the ligands of OX40 and 4-1BB [5–8]. Although GITRL has also been found on endothelial cells and activated T cells, the role is unclear [9]. The GITR/GITRL pathway has been reported to modulate dendritic cell function and promote T-cell mediated immunity [10]. Like most TNFR family members, human GITR-GITRL interaction appears to be trimeric while the murine GITRL binds GITR through a dimeric fashion [11, 12]. Currently, the significance of the differential ligand binding GITR-GITRL between human and murine cells remains largely unclear.

Primary Sjögren's syndrome (pSS) is a systemic autoimmune disease characterized by keratoconjunctivitis sicca, xerostomia, and extraglandular abnormalities [13]. However, the precise etiology remains unclear. At the immunological level, numerous studies have indicated that both T-cell activation and proinflammatory cytokine production are pivotally involved in pSS pathogenesis. Previous studies have demonstrated that the functional interaction of GITR with its cognate ligand GITRL can provide a costimulatory signal to both CD4^+^ and CD8^+^ naive T cells, enhancing proliferation and effector function [3, 6, 14, 15]. There is increasing evidence indicating that GITR and GITRL are involved in the pathogenesis of autoimmune disease. Several studies have reported that the GITR/GITRL pathway plays an important role in autoimmune diseases, as demonstrated by experimental models of experimental autoimmune encephalomyelitis, collagen-induced arthritis and autoimmune diabetes [15–17]. Our recent studies have revealed a close association of increased GITRL expression with disease activity in patients with SLE (Lei et al.) [18]. To determine whether GITR and GITRL system participates in pSS pathogenesis, we measured the serum levels of sGITR and GITRL in pSS patient and investigated correlation of their expression levels with clinical and laboratory variables. Moreover, the expression of the GITR and GITRL in the salivary glands of patients with pSS was also examined by immunohistochemistry.

## 2. Materials and Methods

### 2.1. Patients and Serum Samples

The study group comprised 41 patients (39 women and 2 men) with a mean age of 36.42 ± 18.71 years. All patients were recruited from the Department of Rheumatology, The First Affiliated Hospital of Nanjing Medical University between September 2011 and September 2012 and diagnosed with primary SS fulfilled the American-European Consensus Group Criteria for this diagnosis [19], and individuals with other rheumatic diseases, infections, or malignant tumors were excluded from the study. Sera were also collected from 29 healthy controls at the same hospital, and all recruited healthy controls were excluded from having any autoimmune diseases. There were no significant differences in the ages or sex ratios between the two groups. In addition, labial salivary gland (LSG) biopsy specimens were collected from 10 female patients that matched the histological criteria for a diagnosis of SS [20] and had severe cellular infiltration (focus index ≥ 1). The biopsies were performed for routine diagnostic purposes after obtaining the patient's consent. There were also 6 control LSG specimens from subjects who did not fulfill the classification criteria for pSS but had sicca symptoms, such as dry mouth or dry eye. The controls were matched for sex and age to the pSS patients and had been examined for the presence of rheumatic disease including secondary SS. Both the research protocol and the consent forms were approved by the Research Ethics Committee of Jiangsu Province Hospital.

### 2.2. Clinical and Immunological Data

All patients underwent extensive medical examinations and serological evaluations, including measurements of antinuclear antibodies (ANA), anti-Ro/SSA antibody (A-SSA), anti-La/SSB antibody (A-SSB), erythrocyte sedimentation rate (ESR), C-reactive protein (CRP), immunoglobulin G (IgG), immunoglobulin M (IgM), immunoglobulin A (IgA), and rheumatoid factor (RF). In addition, the numbers of cellular infiltration per 4 mm^2^ of tissue (focus index, FI) were measured. The clinical data from the patients were recorded in Table 1. 

A volume of 5 mL peripheral venous blood was collected from each patient and health control subject and waited to clot at room temperature for 2 hours. Samples were then centrifuged for 10 minutes at 800 g. The serum samples were separated and frozen at −80°C until further analysis.

### 2.3. Labial Salivary Gland Biopsy

LSG biopsies were taken from patients with sicca symptoms. For this, local anesthetic was injected into the lower lip and a small incision to the right or left of the lip midline was made. Four or five LSG lobules were harvested and placed into Carnoy's fixative for 24 hours. Standard paraffin preparations were prepared for sectioning at a thickness of 3 *μ*m and stained with hematoxylin and eosin. The slides were examined for the presence of lymphocytic infiltrates and/or foci using standardized criteria. The focus index (FI) was recorded as the number of foci per 4 mm^2^ of LSG tissues.

### 2.4. Measurement of Serum sGITR and GITRL

Serum sGITR and GITRL levels were measured using ELISA kits (RayBiotech Inc.) according to the manufacturer's protocols. Briefly, serum samples (1 : 50 dilution) and standards were added to the 96-well plate and incubated overnight at 4°C. After incubation for 2 hours and washing 3 times, biotinylated antihuman GITRL antibodies were added, followed by incubation with HRP-conjugated streptavidin and color development with TMB substrate solution. The intensity of the color reaction was measured by a microplate reader (Bio-Rad, Beijing, China) at a wavelength of 450 nm. Concentrations of sGITR and GITRL were determined by a standard curve according to the manufacturer's instructions.

### 2.5. Immunohistochemical Staining for GITR and GITRL

The fixed LSG biopsy specimen slides were fixed in Carnoy's fixative and embedded in paraffin wax. Paraffinized LSG tissues were sectioned to 3 *μ*m thickness, deparaffinized in xylene, and rehydrated through a series of concentrations of ethanol. After inactivation of endogenous peroxidase, sections were blocked by incubation with 5% bovine serum album for 30 minutes at room temperature then incubated with either rabbit anti-human GITRL (Santa Cruz) or mouse anti-human GITR (R&D Systems) at 4°C overnight in a humidified chamber and both of which were diluted 1 : 50. The slides were washed for 5 minutes; sections were then incubated with peroxidase-conjugated goat anti-rabbit or goat anti-mouse secondary antibody for 1 hour at room temperature; both of which were diluted 1 : 200. The reactions were developed using a DAB substrate kit, with hematoxylin as counterstain. Each slide was evaluated by one of the authors (Ms. Xiaoke Feng) under a microscope (Nikon, Tokyo, Japan). 

### 2.6. Statistical Analysis

Data were presented as mean ± standard deviation unless specified otherwise. Statistical analysis was performed using SPSS for Windows (version 11.5). The Mann-Whitney rank sum test or Kruskal-Wallis tests were used to compare sGITR and GITRL levels. The correlation between GITRL/sGITR levels and various values were analyzed by Spearman's rank correlation coefficient. A value of *P* < 0.05 was considered statistically significant.

## 3. Results

### 3.1. Primary SS Patients Showed Higher Serum GITRL and sGITR Levels Than Healthy Controls

**Figure 1:**
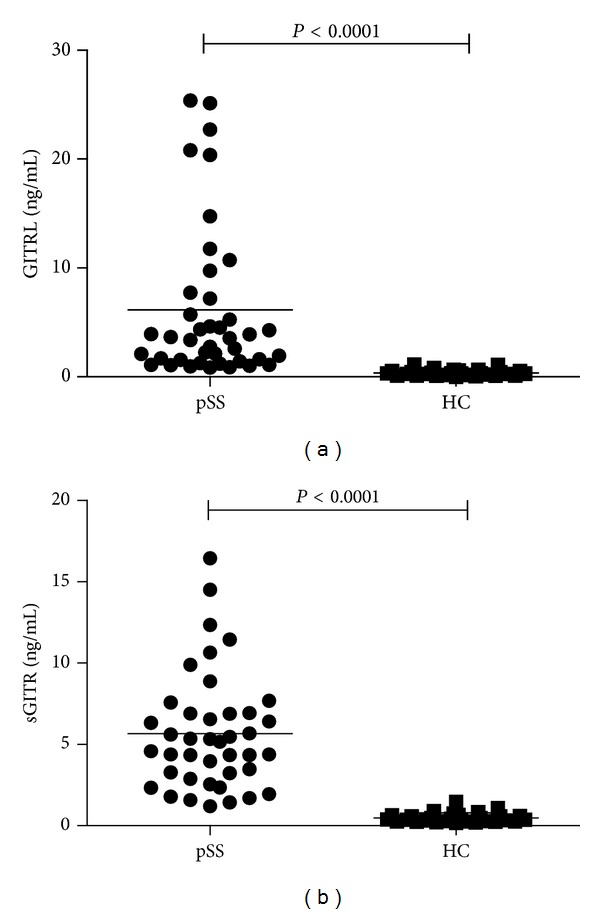
Comparison of serum GITRL and sGITR levels between pSS and HC. (a) Serum GITRL levels were significantly elevated in SS patients versus HC. (b) Serum sGITR levels were also significantly elevated in SS patients versus HC. Each symbol represents an individual patient and healthy donor. Horizontal lines indicate median values. GITRL: glucocorticoid-induced TNFR-related protein ligand; sGITR: soluble glucocorticoid-induced TNFR-related gene; SS: Sjögren's syndrome; HC: healthy control.

Serum GITRL and sGITR levels in pSS (*n* = 41) and healthy controls (*n* = 29) were measured by ELISA analysis. As shown in Figure 1, serum levels of GITRL were significantly higher in pSS patients than in HC (6.17 ± 7.10 ng/mL versus 0.36 ± 0.28 ng/mL; *P* < 0.0001). In addition, serum sGITR levels were also markedly elevated in pSS patients when compared with HC (5.66 ± 3.56 ng/mL versus 0.50 ± 0.31 ng/mL; *P* < 0.0001). The results suggest that GITRL and sGITR overexpression is possibly involved in the pathogenesis of pSS.

### 3.2. Serum GITRL and sGITR Levels Were Markedly Higher in Primary SS Patients with Extraglandular Manifestations

**Figure 2:**
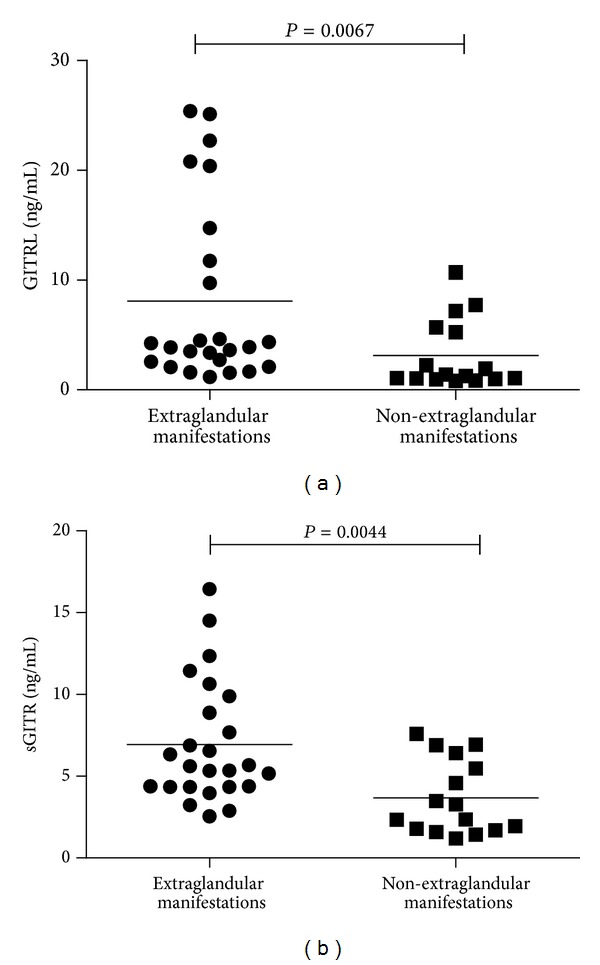
Comparison of serum GITRL and sGITR levels between pSS patients with or without extra-glandular manifestations. (a) Serum GITRL levels exhibited elevation in pSS patients with extra-glandular manifestations (*n* = 25) relative to patients without extra-glandular manifestations (*n* = 16). (b) Serum sGITR levels were also higher in pSS patients with extra-glandular manifestations (*n* = 25) than patients in the absence of extra-glandular manifestations (*n* = 16). Each symbol represents an individual patient; horizontal lines indicate median values.

We further divided pSS patients into groups with or without extraglandular manifestations according to their symptoms. pSS patients with sicca symptoms only such as dry mouth or dry eye were defined as the non-extraglandular manifestations group, while other patients with fever, arthritis, anemia, leukopenia, thrombocytopenia, renal disease, pulmonary interstitial changes, or autoimmune liver dysfunction were extraglandular manifestations group. As shown in Figure 2, both serum GITRL and sGITR levels in pSS patients with extra-glandular manifestations were significantly higher than those without extra-glandular manifestations (GITRL: 8.60 ± 8.25 ng/mL versus 3.15 ± 3.13 ng/mL; *P* = 0.0067. sGITR: 6.92 ± 3.70 ng/mL versus 3.68 ± 2.27 ng/mL; *P* = 0.0044). 

### 3.3. Elevated Serum GITRL and sGITR Levels Were Associated with Increased Foci Index (FI) in Primary SS Patients

**Figure 3:**
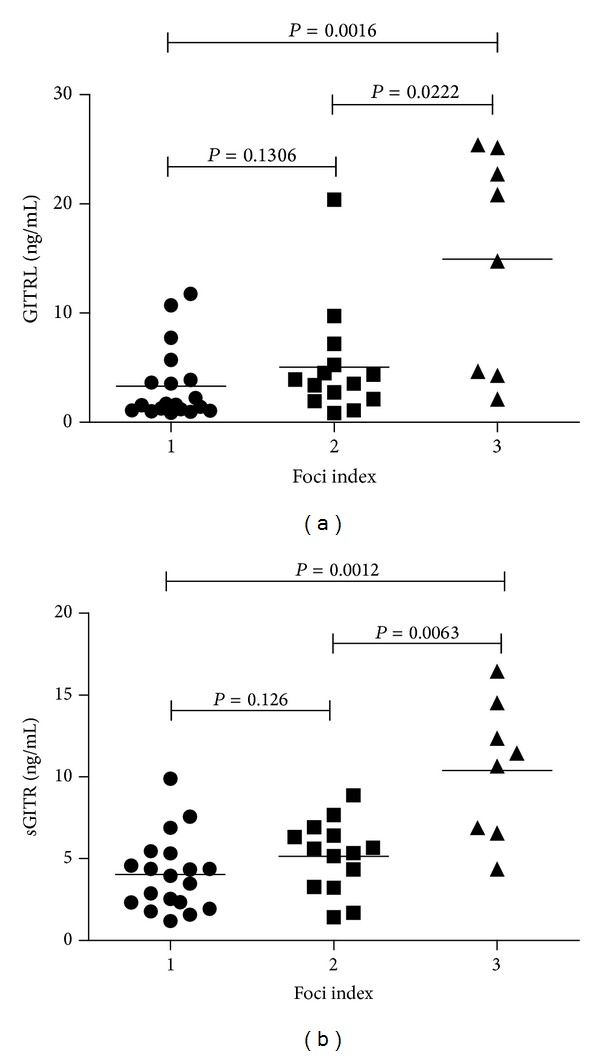
Comparison of serum GITRL and sGITR levels among pSS patients with different foci index. pSS patients were divided according to the foci index (1~3) in labial salivary glands (LSGs) of lymphocytic infiltration (see Methods). (a) Serum GITRL levels were significantly higher in pSS patients with serious sialadenitis. (b) Serum sGITR levels were also elevated as foci index increased in pSS patients.

The FI was recorded as the number of foci per 4 mm^2^ of LSGs. We divided pSS patients into various groups according to their FI. As shown in Figure 3, elevated GITRL and sGITR levels were closely associated with increased severity of lymphocytic infiltration. Serum GITRL levels were significantly higher in FI 3 group of pSS patients (FI 3 versus FI 2: 14.97 ± 9.95 ng/mL versus 5.16 ± 5.20 ng/mL; *P* = 0.0222. FI 3 versus FI 1: 14.97 ± 9.95 ng/mL versus 3.31 ± 3.33 ng/mL; *P* = 0.0016). Similarly, serum sGITR also markedly elevated in FI 3 group (FI 3 versus FI 2: 10.39 ± 4.18 ng/mL versus 5.19 ± 2.16 ng/mL; *P* = 0.0063. FI 3 versus FI 1: 10.39 ± 4.18 ng/mL versus 4.04 ± 2.27 ng/mL; *P* = 0.0012). However, the FI 2 group did not differ significantly from the FI 1 group.

### 3.4. Correlation of Serum GITRL and sGITR Levels with Other Laboratory Observations

**Figure 4:**
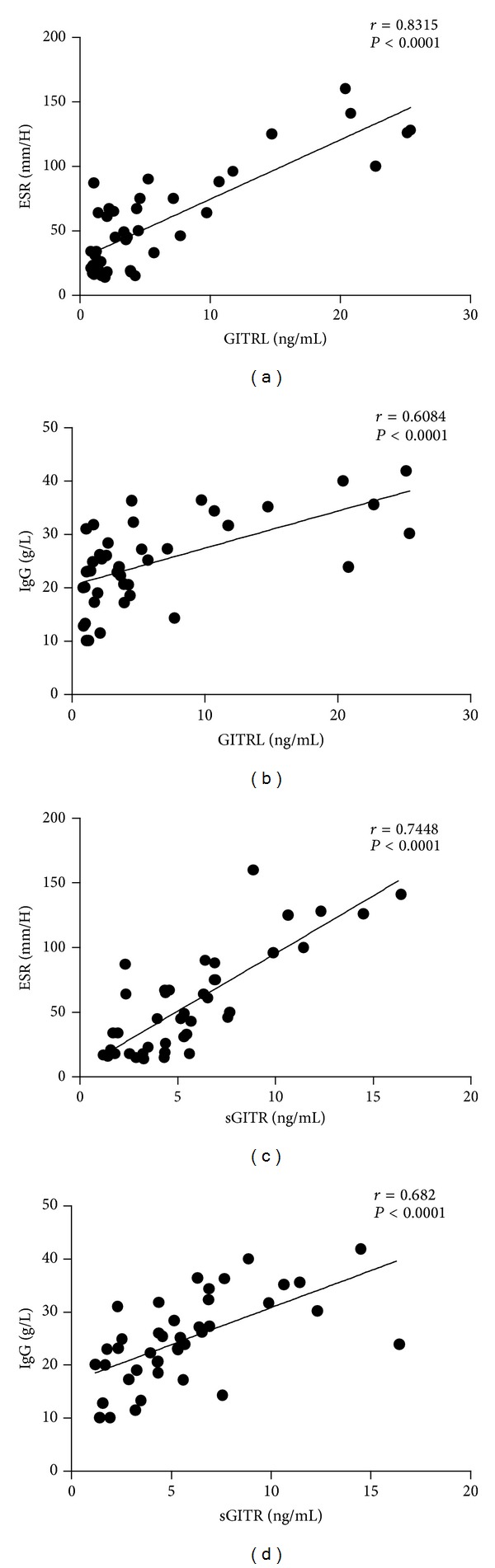
Correlation of serum GITRL and sGITR levels with laboratory values. (a), (b) Positive correlation was observed between serum GITRL levels and ESR, IgG. (c), (d) Positive correlation was also seen between serum sGITR levels and ESR, IgG. ESR: erythrocyte sedimentation rate; IgG: immunoglobulin G.

To further determine the relationship between serum GITRL/sGITR levels and laboratory test results including the titers of ANA, A-SSA, A-SSB, ESR, CRP, RF, and Ig levels, it was found that both serum GITRL and sGITR levels were positively correlated with ESR (GITRL: *r* = 0.8315, *P* < 0.0001; sGITR: *r* = 0.7448, *P* < 0.0001, Figures 4(a) and 4(c)) and IgG (GITRL: *r* = 0.6084, *P* < 0.0001; sGITR: *r* = 0.6820, *P* < 0.0001, Figures 4(b) and 4(d)). However, no significant correlations were found between serum GITRL or sGITR levels and the other laboratory values (data not shown). Interestingly, when patients were grouped according to test results normal or abnormal, elevated serum GITRL levels exhibited were found in the groups with high titers of ANA (6.85 ± 7.32 versus 1.22 ± 0.42, *P* = 0.0050) or high concentration of CRP (15.87 ± 8.76 versus 4.41 ± 4.75, *P* = 0.0078), RF (8.98 ± 8.64 versus 2.47 ± 1.72, *P* = 0.0050), IgM (10.48 ± 8.96 versus 5.01 ± 6.30, *P* = 0.0431), and IgA (7.93 ± 7.92 versus 2.34 ± 1.94, *P* = 0.0069), while serum sGITR levels were significantly elevated in the patients with high titers of ANA (6.09 ± 3.58 versus 2.51 ± 0.83, *P* = 0.0114) or high concentration of CRP (7.05 ± 3.60 versus 4.76 ± 3.305, *P* = 0.0231), RF (6.93 ± 3.95 versus 3.85 ± 1.85, *P* = 0.0075), and IgA (6.48 ± 3.81 versus 3.87 ± 2.14, *P* = 0.0381). In addition, patients with leukopenia were observed having markedly increased serum GITRL and sGITR levels (GITRL: 9.18 ± 8.91 versus 3.29 ± 2.96, *P* = 0.0158; sGITR: 7.28 ± 4.03 versus 4.10 ± 2.18, *P* = 0.0110), as shown in Table 2. 

### 3.5. High Expression of GITRL and GITR in the Labial Salivary Glands (LSGs) from Primary SS Patients

**Figure 5:**
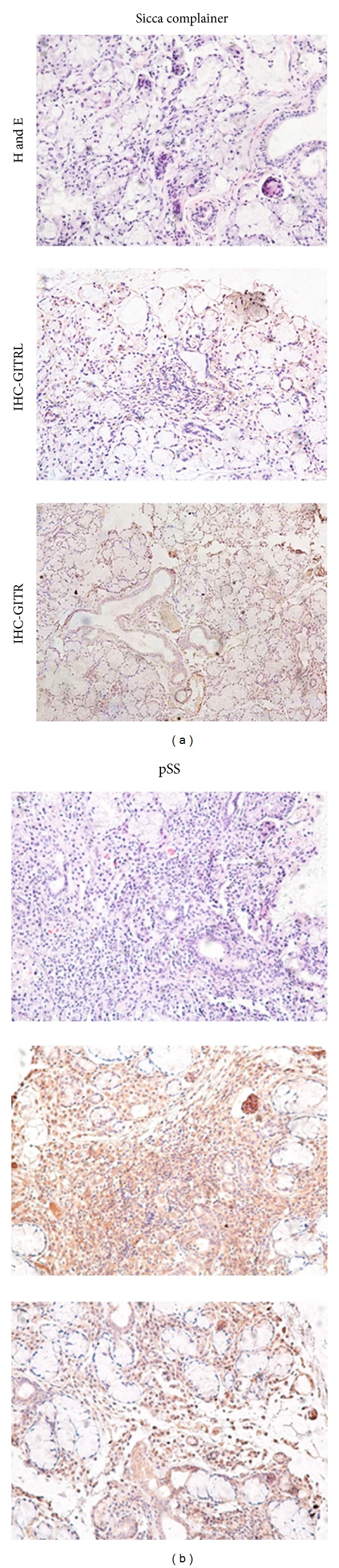
High expression of GITRL and GITR in labial salivary glands from patients with pSS. The labial salivary glands (LSG) of pSS patients exhibit increased GITRL and GITR expression. Shown is the expression of the GITRL and GITR in the labial salivary glands of sicca complainer (*n* = 6, left column) and patients with pSS (*n* = 10; right column), as determined by immunostaining using specific antibodies. The cells that stained with the antibodies appeared in brown. The infiltrating lymphocytes and periductal areas of LSG from the patients with pSS exhibited intense GITRL and GITR staining, whereas the expression of GITRL was stronger and more widely distributed than GITR. In contrast, the sicca complainers did not exhibit any GITRL or GITR expression in their labial salivary glands.

To evaluate the local effect of GITRL and GITR in LSGs, we applied immunohistochemical staining to determine the expression of GITRL and GITR in LSGs. The freshly explanted lower lip biopsy specimens were sectioned and stained with anti-GITRL and anti-GITR antibodies. All 10 pSS samples exhibited distinct expression of GITRL, while 6 of the 10 samples were positive for GITR. In contrast, none of the LSGs from the sicca complainers exhibited GITRL or GITR expression. Six specimens from pSS patients stained for GITR showed similar pattern of GITRL and GITR expression; that is, GITRL was prominent in infiltrating lymphocytes and ductal cells, while GITR was mainly expressed in infiltrating lymphocytes with a weak expression observed on ductal cells. Overall, the expression of GITRL was stronger and more widely distributed than GITR. Both GITRL and GITR were expressed on lymphocytic infiltrates and to a lesser degree in the acinar components (Figure 5).

## 4. Discussion

In this study, we determined the serum levels of sGITR and GITRL in pSS patients and further revealed the positive correlation of their expression levels with IgG and ESR, which were in association with disease severity of pSS. In addition, we have shown for the first time the expression pattern of GITR and GITRL in the salivary glands of patients with pSS. 

Several studies including our recent findings have suggested that GITR-GITRL system is involved in the pathogenesis of autoimmune disease including rheumatoid arthritis (RA) and SLE [18, 21]. In several mouse autoimmune disease models including experimental autoimmune encephalomyelitis, collagen-induced arthritis, inflammatory bowel diseases, gastritis, thyroiditis, and autoimmune diabetes, GITR inhibition resulted in an anti-inflammatory effect. In accordance, GITR triggering by GITRL or anti GITR antibody exerted an increased inflammatory response [15–17, 22–24]. GITR and GITRL system also participates in the development of autoimmune diseases in human as demonstrated in RA recently, which was found that GITRL protein levels in the serum samples of RA patients were significantly higher than those in samples from healthy control subjects. Furthermore, the increased levels of GITRL in RA patients were positively correlated with the DAS-28 scores of these patients [21]. Recently, we have reported that serum GITRL levels are markedly increased in patients with SLE compared with healthy controls, especially in patients with active disease. Moreover, serum GITRL levels are found to be positively correlated with SLEDAI, titers of anti-dsDNA antibody, ESR, and IgM but negatively correlated with C3. In addition, serum GITRL levels elevated in SLE patients with renal involvement and vasculitis compared with patients that the above-mentioned manifestations were absent [18]. However, the role of GITR-GITRL system in the pathogenesis of SS is poorly understood. Saito et al. have demonstrated that Sjögren's syndrome-like autoimmune sialadenitis in MRL-Fas^lpr^ mice was associated with expression of GITRL in salivary gland duct epithelial cells [25], consistent with our current observations. Together, these results indicate a proinflammatory role of GITR/GITRL pathway in driving autoimmune progression in various autoimmune diseases. 

Recent studies have suggested that in most of inflammatory and autoimmune diseases, the activation of GITR-GITRL pathway promotes leucocyte extravasation, increases T lymphocyte activation, and partially reverses the immunosuppressive function of Treg [26–28]. Several lines of evidence support the notion that the insufficiency or dysfunction of Treg may contribute to the breakdown of immune tolerance, leading to the procession of autoimmune diseases along with the interruption of immune homeostasis [29–31]. Since previous studies have reported that the numbers of Treg are positively correlated with higher grade of infiltration at the salivary glands in pSS [32], we speculate that GITR/GITRL activation may participate in pSS pathogenesis by inhibiting Treg immunosuppressive activity and increasing T lymphocyte activation. It has been reported that signaling downstream of GITR/GITRL pathway results in the activation of NF-*κ*B as well as the members of the MAPK pathway including p38, JNK, and ERK [1, 3, 33], which in turn enhances T cell survival by upregulating IL-2Ra, IL-2, and IFN-*γ* [3]. Further studies are warranted to investigate how GITR/GITRL pathway modulates the homeostatic regulation of Treg/Th17 cells during the pathogenesis of autoimmune diseases.

In this study, we have demonstrated a close correlation of serum elevated GITRL and sGITR with the increased degree of lymphocytic infiltration in patients with pSS. Moreover, GITR and GITRL were readily detected in the lymphocytic foci and periductal areas of the LSGs. In contrast, the LSGs of healthy control subjects did not express GITR or GITRL. Moreover, we have further detected a sharp decrease of the stimulated salivary flow in the pSS patients with glandular cells positively stained for GITRL (data not shown). Our findings suggest that GITR and GITRL may play an important role in the sialadenitis suffered by patients with pSS. In addition, our investigations have revealed a close correlation of circulating sGITR and GITRL levels with the disease activity and severity in pSS patients. Our data have clearly shown that serum levels of sGITR and GITRL positively correlate with ESR and IgG, and exhibit elevation in the groups with extra-glandular manifestations as well as abnormal laboratory parameters such as ANA, CRP, IgM, IgA, and WBC. We provide new evidence indicating involvement of GITR/GITRL overactivation in the disease pathophysiology of pSS, which may serve as a new biomarker to assess the disease activity and severity of pSS.

## 5. Conclusion

Our findings indicate the possible involvement of GITR-GITRL pathway in the pathogenesis of pSS. Further investigations on the systemic and localized effects of GITR and GITRL in pSS may facilitate the development of targeting this molecule pathway for the treatment of pSS.

## Figures and Tables

**Table 1:** Characteristics of pSS patients and the control subjects.

	pSS (*n* = 41)	Control (*n* = 29)
Age (years)	36.42 ± 18.71	34.53 ± 21.47
Sex (female/male)	39/2	28/1
Disease duration (months)	36.33 ± 42.25	—
Arthritis (%)	6 (14.63)	—
Fever (%)	8 (19.51)	—
Anemia (%)	12 (29.27)	—
Leukopenia (%)	20 (48.78)	—
Thrombocytopenia (%)	4 (9.76)	—
Renal disease (%)	7 (17.07)	—
Pulmonary interstitial changes (%)	5 (12.20)	—
Autoimmune liver disfunction (%)	4 (9.76)	—
ANA positive (%)	36 (87.80)	—
A—SSA positive (%)	34 (82.93)	—
A—SSB positive (%)	19 (46.34)	—
ESR (mm/H)	56.75 ± 39.38	—
CRP (mg/L)	4.86 ± 3.21	—
IgG (g/L)	24.76 ± 8.12	—
IgM (g/L)	1.97 ± 1.87	—
IgA (g/L)	3.76 ± 1.51	—
RF (IU/L)	67.79 ± 32.47	—
Focus index (1–3)	1.56 ± 0.77	—

pSS: primary Sjögren's syndrome; ANA: antinuclear antibody; A-SSA: anti-Ro/SSA antibody; A-SSB: anti-La/SSB antibody; ESR: erythrocyte sedimentation rate; CRP: C-reactive protein; IgG: immunoglobulin G; IgM: immunoglobulin M; IgA: immunoglobulin A; RF: rheumatoid factor; Focus index: the number of foci per 4 mm^2^ of tissue.

Values are expressed as mean ± standard deviation.

**Table 2:** Comparison of serum GITRL and sGITR levels between pSS patients with normal or abnormal laboratory values.

Parameter	GITRL (ng/mL)			sGITR (ng/mL)		
	Normal	Abnormal	*P* value	Normal	Abnormal	*P* value
	Mean ± SD (*n*)	Mean ± SD (*n*)		Mean ± SD (*n*)	Mean ± SD (*n*)	
A—SSA	3.23 ± 3.63 (7)	6.76 ± 7.52 (34)	0.1362	3.91 ± 2.14 (7)	6.01 ± 3.71 (34)	0.1657
A—SSB	6.41 ± 7.38 (22)	5.95 ± 7.03 (19)	0.5830	5.71 ± 3.57 (22)	5.60 ± 3.63 (19)	0.8857
ANA	1.22 ± 0.42 (5)	6.85 ± 7.32 (36)	**0.0050	2.51 ± 0.83 (5)	6.09 ± 3.58 (36)	*0.0114
CRP	4.41 ± 4.75 (25)	15.87 ± 8.76 (16)	**0.0078	4.76 ± 3.30 (25)	7.05 ± 3.60 (16)	*0.0231
RF	2.47 ± 1.72 (17)	8.98 ± 8.64 (24)	**0.0050	3.85 ± 1.85 (17)	6.93 ± 3.95 (24)	**0.0075
IgM	5.01 ± 6.30 (33)	10.48 ± 8.96 (8)	*0.0431	5.31 ± 3.39 (33)	7.08 ± 4.13 (8)	0.2428
IgA	2.34 ± 1.94 (13)	7.93 ± 7.92 (28)	**0.0069	3.87 ± 2.14 (13)	6.48 ± 3.81 (28)	*0.0381
WBC	3.29 ± 2.96 (21)	9.18 ± 8.91 (20)	*0.0158	4.10 ± 2.18 (21)	7.28 ± 4.03 (20)	*0.0110

*P* < 0.05 means significant difference (*P < 0.05; **P < 0.01).

Laboratory values such as A-SSA, A-SSB, ANA, and RF positive were defined as abnormal, while laboratory parameters such as CRP, IgM, and IgA above limit values were defined as abnormal. In addition, WBC abnormal means leukopenia.

A-SSA: anti-Ro/SSA antibody; A-SSB: anti-La/SSB antibody; ANA: antinuclear antibody; CRP: C-reactive protein; RF: rheumatoid factor; IgM: immunoglobulin M; IgA: immunoglobulin A; WBC: white blood count.
